# On the Accurate Determination of Shock Wave Time-Pressure Profile in the Experimental Models of Blast-Induced Neurotrauma

**DOI:** 10.3389/fneur.2018.00052

**Published:** 2018-02-06

**Authors:** Maciej Skotak, Eren Alay, Namas Chandra

**Affiliations:** ^1^Department of Biomedical Engineering, New Jersey Institute of Technology, Newark, NJ, United States

**Keywords:** shock wave, shock tube, peak overpressure, impulse, sampling frequency, electrostatic charges, baseline drift

## Abstract

Measurement issues leading to the acquisition of artifact-free shock wave pressure-time profiles are discussed. We address the importance of in-house sensor calibration and data acquisition sampling rate. Sensor calibration takes into account possible differences between calibration methodology in a manufacturing facility, and those used in the specific laboratory. We found in-house calibration factors of brand new sensors differ by less than 10% from their manufacturer supplied data. Larger differences were noticeable for sensors that have been used for hundreds of experiments and were as high as 30% for sensors close to the end of their useful lifetime. These observations were despite the fact that typical overpressures in our experiments do not exceed 50 psi for sensors that are rated at 1,000 psi maximum pressure. We demonstrate that sampling rate of 1,000 kHz is necessary to capture the correct rise time values, but there were no statistically significant differences between peak overpressure and impulse values for low-intensity shock waves (Mach number <2) at lower rates. We discuss two sources of experimental errors originating from mechanical vibration and electromagnetic interference on the quality of a waveform recorded using state-of-the-art high-frequency pressure sensors. The implementation of preventive measures, pressure acquisition artifacts, and data interpretation with examples, are provided in this paper that will help the community at large to avoid these mistakes. In order to facilitate inter-laboratory data comparison, common reporting standards should be developed by the blast TBI research community. We noticed the majority of published literature on the subject limits reporting to peak overpressure; with much less attention directed toward other important parameters, i.e., duration, impulse, and dynamic pressure. These parameters should be included as a mandatory requirement in publications so the results can be properly compared with others.

## Introduction

The blast-induced traumatic brain injury (bTBI) is a signature “invisible wound” among active military personnel partaking in war theaters in Iraq and Afghanistan (1–3). Specifically, the mild TBI (mTBI) remains an important public health problem: 2000–2016 period, the Department of Defense reported more than 360,000 cases of combat- and non-combat-related head injuries of which 82.4% were mTBI (4, 5). A recent analysis of literature related to studies performed on veterans returning from deployment indicates the “at least half of the TBIs related to the wars in Iraq and Afghanistan appears to be blast related” (6). Human studies provide an array of health outcomes associated with mTBI, ranging from anxiety, depression, post-traumatic stress disorder, and post-concussive symptoms (7), but it is difficult to correlate precisely these symptoms with exposure to blast without knowledge about levels and frequency of exposure. This situation was partially ameliorated by the deployment of Blast Gauge™ system (8), which aids in the retrieval of this crucial information about overpressure “dosage” (a waveform exceeding the predefined threshold is recorded with 100 kHz sampling frequency) and in turn, allows reconstruction of specific incidents involving blasts (9). A large amount of collected data, issues with data interpretation and lack of associated medical history has inhibited the development of correlations between exposure levels with outcomes (10).

Animal models are, thus used to gain an insight into etiology of brain trauma and to study underlying molecular mechanism. The success of these studies in bTBI research area relies on a number of factors, and the two chief ones are: (1) appropriate exposure condition, equivalent to those experienced in the field and (2) proper animal restraint to eliminate injuries other than those classified as primary bTBI (exposure to shock wave only) (11, 12). In the laboratory setting, the shock tubes are used to re-create bTBI etiology in animal models (13–15). The importance of animal location on the characteristics of the loading conditions has been recently demonstrated (16): in general, animals mounted for testing inside of the shock tube are exposed to shock wave resulting in primary blast TBI (17). Animal models tested inside experience a higher level of loading caused by static pressure, simply because higher peak overpressures and durations can be achieved inside when compared with the outside. This difference is demonstrated in reported durations in the published literature where unlike inside the shock tube, overpressure durations longer than 2 ms have not been reported outside of the shock tube (18–22). Furthermore, acceleration of the rodent head, and body caused by dynamic pressure (jet effect) of expanding shock wave (classified as tertiary blast injury) are characterized by increased level of injury compared with primary blast TBI (23).

The distinction between primary and other types of TBI is important to allow comparison of results between laboratories. However, this comparison will not be complete if shock wave-associated parameters are not measured and disclosed in the reports. We have screened a pool of 100 papers selected from existing literature on animal and *in vitro* models of bTBI covering the last 20 years of research to identify reporting standards regarding shock wave parameters. This survey gives an overview and revealed the following trends: in a majority of the published work (97%), peak overpressure is reported as the most important parameter (see Table S1 in Supplementary Material for details). Interestingly, little attention is devoted to other characteristics of the shock wave waveform: the duration (51%), impulse (18%), the sampling frequency (46%), and finally the rise time, which is essentially neglected (2%). Only in very few papers, all four of these parameters were provided (20, 24–27), including or not sampling rates used in these studies.

In this paper, we discuss issues related to overpressure measurements: the importance of pressure transducer calibration, the effect of sampling frequency on the quantification of shock waveform characteristics, and preventive measures to avoid signal corruption. Electromagnetic interference and mechanical vibration are two aggravating factors that lead to an acquisition of incorrect waveforms with exaggerated peak overpressure values and baseline drifts, which might lead to erroneous duration and impulse values. We present preventive measures, which are illustrated with examples of experimental data.

## Materials and Methods

### The Shock Tubes

**Figure 1:**
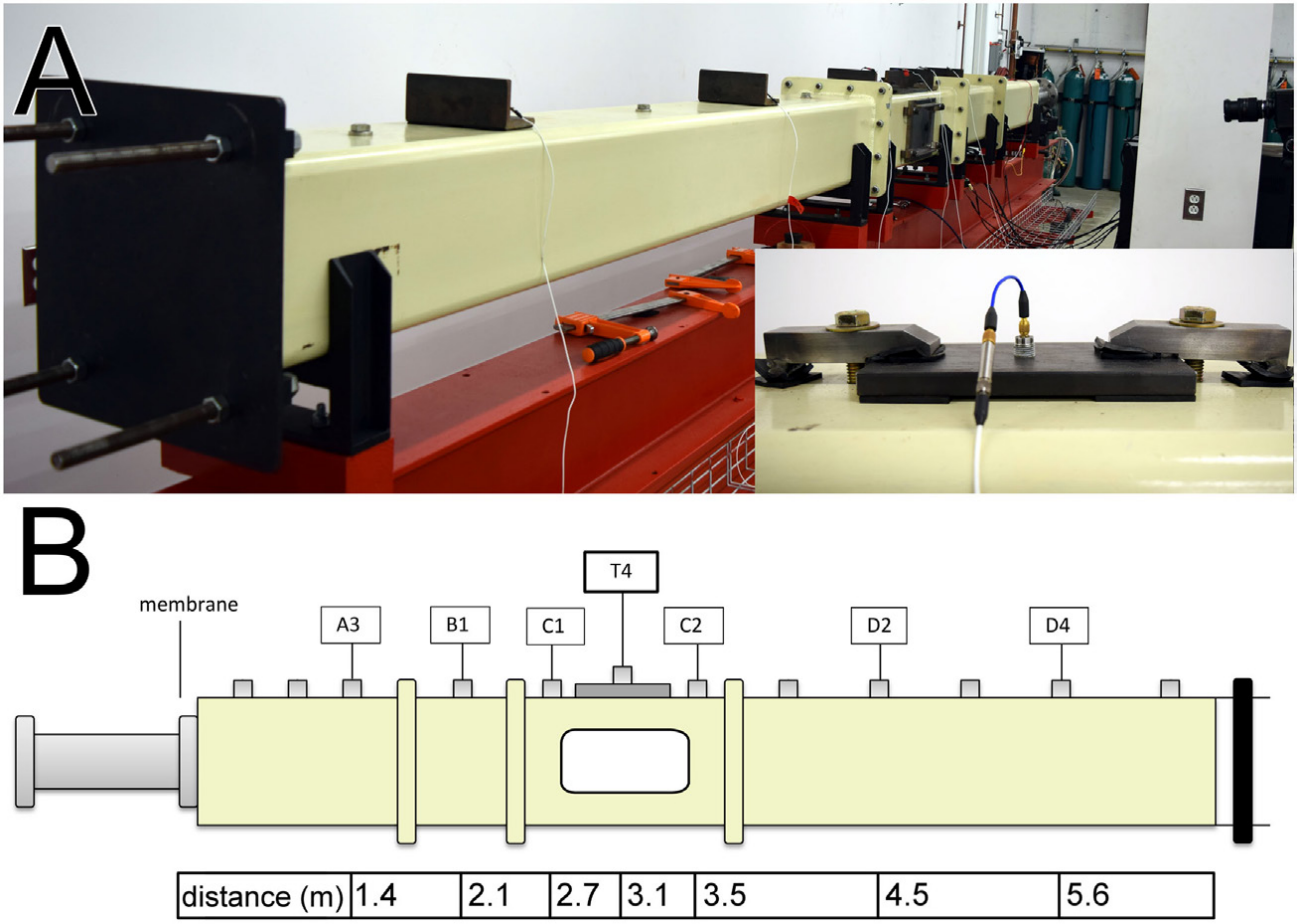
The 9-inches square cross section shock tube: the rear view with the reflector plate and sensor brackets distributed along the shock tube **(A)**. The inset depicts PCB 134A24 sensor (connected *via* 3 in long low-noise cable to a series 402 charge amplifier) in the modified sensor mounting system with rubberized brackets and steel clamps preventing vertical movement of the sensor caused by the pressure of the passing shock wave. Schematic representation of the shock tube with sensor labeling and sensor distances from the membrane mounting port in the breech **(B)**. Only ports used in experiments described therein are indicated in the diagram.

Both large (28-inch square cross section) and small (9-inch square cross section) scale shock tubes at the New Jersey Institute of Technology have modular design and the following characteristics (Figure 1): (1) adjustable volume breech, (2) variable length transition section, (3) the 6 m long test section, equipped with bullet-proof glass windows for high-speed video observation of the specimen during the shock wave exposure, and (4) the reflector end-plate. In all experiments described in this contribution, the compressed helium was filled into the fixed volume breech, which is separated from the main body of the shock tube by Mylar membranes [for a detailed description of the principles of operation an informed reader is referred to Ref. (28, 29)].

### Overpressure Measurement

A series of pressure sensors distributed along the length of the shock tube was used to measure pressure-time profiles of incident shock wave (Figure 1B) (28). The incident pressure was measured using high-frequency Tourmaline pressure transducers model 134A24 (1,000 psi maximum pressure, resonant frequency ≥1,500 kHz, 0.2 μs rise time, PCB Piezotronics, Depew, NY, USA). These sensors use 402 charge amplifiers, which are connected in series with pressure transducers *via* 3-inch-long low-noise cable with 10–32 coaxial jacks. In headform exposure experiments, we used high-frequency ICP^®^ model 102B06 pressure transducers (500 psi maximum pressure, resonant frequency ≥500 kHz, and ≤1.0 μs rise time, PCB Piezotronics, Depew, NY, USA). All data were recorded at 1.0 MHz sampling frequency, and the typical acquisition time ranged from 50 to 200 ms. The 134A series sensors have 1/2–20 thread and require mounting holes of larger diameter; we used 5/8–18 tapped threaded holes, which are blinded with appropriate screws when not in use. Sensors are mounted in 0.25-inch thick steel brackets with rubber feet to prevent the transfer of the vibrations from the shock tube wall. Brackets with sensors are clamped to the shock tube wall, which prevents their displacement by pressure during shock wave experiments (Figure 1A, inset). There are 11 pressure sensor mounting openings in the shock tube designated as An, Bn, Cn, or Dn, depending on which module of the shock tube these sensors are located, where the number *n* = 1, 2, 3,… indicates the consecutive sensor count for that specific module (Figure 1B). There are an additional nine ports in the test section located in the C module of the shock tube and denoted as *Tn* (for *n* = 1–9). Typically, only six to seven sensors are used at the same time to capture a representative snapshot of the evolution of the shock wave profile.

### Pressure Sensor Calibration

Pressure sensor calibration methodology developed in our lab relies on the measurement of the shock wave velocity which is related to the overpressure using Rankine–Hugoniot relationship:

(1) $$\Delta P=\frac{\left(M^{2}-1\right)2\gamma_{i}P_{at}}{\gamma_{i}},$$

where Δ*P*—overpressure, *M*—Mach number, γ*_i_*—specific heat ratio (1.4 for nitrogen, 1.66 for helium), *P*_at_—atmospheric pressure.

**Figure 2:**
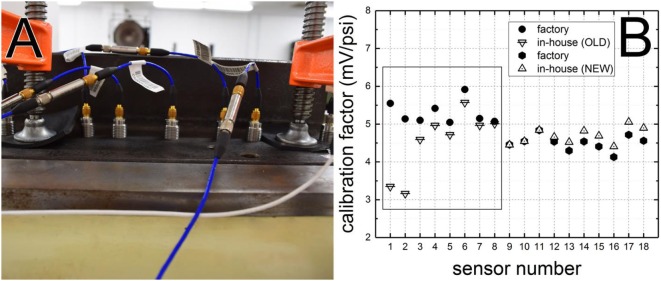
An array of seven pressure transducers (PCB 134A24) mounted on a plate in the test section and ready for calibration shots **(A)**. The plate is protected against vertical displacement using two clamps with rubber padding. Comparison of the factory calibration factors and those obtained using experiments performed in-house **(B)**. Data for sensors with long experimental life are outlined with a black rectangle (sensor nos. 1–8). Discrepancies in calibration factor values of below 5% were noted for brand new sensors (sensor nos. 9–18). Pressure transducers are connected *via* 3 in long low-noise cable to the charge amplifier *via* 10–32 jacks, and then *via* long low-noise cable to the data acquisition system.

Typically, an array of seven sensors is mounted in a straight-line along the shock tube axis spanning the distance of 10 inches (first to last, Figure 2A). Sensors are mounted using tapped holes in a single steel plate and mounted in the test section and the plate is secured in place using clamps, to prevent vertical displacement. This is an important precaution for accurate measurement of the sensor output (in volts) which is used to calculate calibration constant. The shock wave velocity is calculated based on the arrival time between sensors and typically a combination of 21 measurements is used. The average velocity is used to calculate corresponding experimental overpressure, Δ*P* from Eq. 1. The voltage output from the sensor divided by Δ*P* gives calibration factor.

### Electrostatic Interference

The baseline drift caused by electrostatic charges was recorded using six pressure sensors (PCB 134A24) distributed along the 9-inch cross section shock tube. Silicone grease (Dow Corning, four Electrical Insulating Grease, McMaster-Carr, cat. no. 1204K12) was purchased and applied to the sensing surface of the sensors to eliminate the baseline drift.

The electrostatic charges leaking into the data acquisition system were observed in experiments with the headform instrumented with 10 PCB 102B06 sensors. These sensors are mounted flush with the surface of the headform and signal cabling is extending from the interior of the headform through the metallic plate (base), the neck and then is lead on the outside of the shock tube. We have routinely observed signal corruption in the event of compromised electrical insulation of the coaxial signal cable caused by shock wave impact and loosening of connector nut. This was also investigated using a dummy sensor setup. Briefly, a 1/4-inch stainless steel tubing was insulated with electrical insulation tape and inserted in the bolt. The entire assembly was mounted in the A3 port in the shock tube (Figure S1 in Supplementary Material), and connected to the data acquisition system.

### The Effect of Sampling Frequency

**Figure 3:**
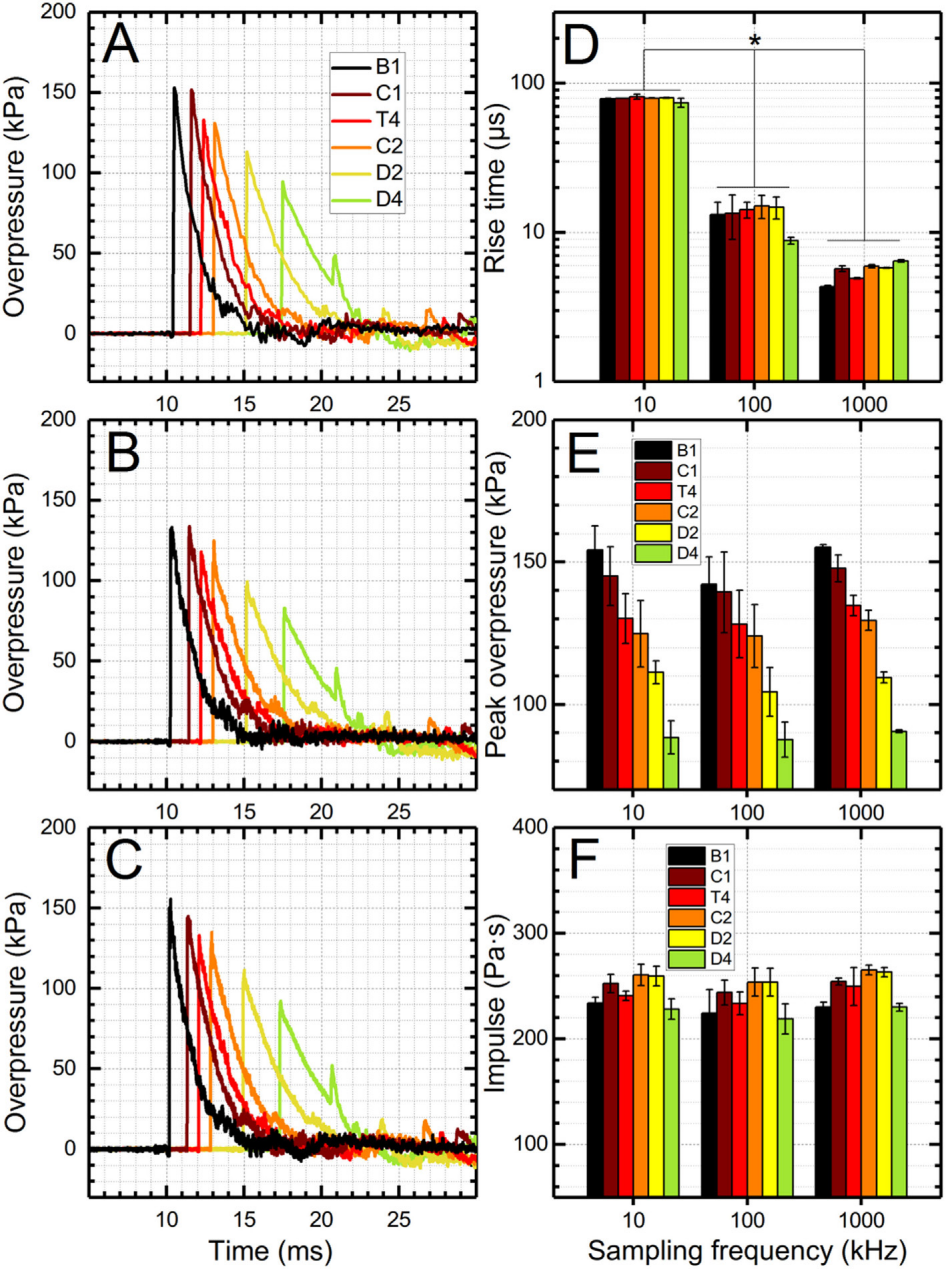
The effect of sampling frequency on the quality of shock wave pressure profiles. Representative pressure profiles recorded using a sampling frequency of 10 kHz **(A)**, 100 kHz **(B)**, and 1,000 kHz **(C)**. Rise time values calculated from respective data sets depend strongly on the sampling frequency **(D)**, while peak overpressure and impulse values are independent of the sampling frequency **(E,F)**, for sampling frequencies used in this study. Statistical significance between respective groups is marked with asterisk (*p* < 0.05).

The effect of sampling frequency on characteristic parameters of the shock wave was evaluated using three sampling frequencies: 10, 100, and 1,000 kHz (Figure 3). A single sampling frequency was set in the DAQ and all experiments were performed using a single shock wave with a nominal intensity of 130 kPa in the test section (T4 sensor, Figure 1B). All tests were repeated four times and the signal was recorded by six pressure sensors located along the shock tube. A total of 72 waveforms was analyzed (3 × 4 × 6 = 72), which included quantification of peak overpressure, rise time, duration, and impulse.

### Sensor Misalignment

The effect of sensor misalignment with respect to the shock tube wall on the pressure profile was tested using sensor located in a T4 position in two configurations: (1) sensor was protruding by 0.25 inch into the shock tube and (2) sensor was receded from the flush position by 0.25 inch. A single shock wave with 130 kPa nominal peak overpressure was generated and compared with an adjacent sensor (denoted as T1, located five inches downstream of the T4 sensor). All measurements were repeated four times and peak overpressure was quantified.

### Statistical Analysis

Data from experiments performed at different experimental conditions were pooled together and checked for normality using Ryan–Joiner test (similar to Shapiro–Wilk) in Minitab 17.0 (Minitab Inc., State College, PA, USA). Then a multiple comparison two-tailed *t*-test (statistical significance threshold was set at *p* < 0.05) was performed with Bonferroni correction on data for evaluation of the effect of sampling frequency and sensor misalignment. All data are presented as a mean and SD.

## Results and Discussion

### Sensor Calibration

The accurate measurements of shock waves rely solely on the characteristics of the sensing element in the pressure transducer, appropriate calibration, and sampling frequency [details of pressure profile evolution in the shock tube are discussed extensively in Ref. (28)]. Tourmaline is among the most popular materials employed as a sensing element in pressure transducers. Its piezoelectric properties were recognized more than 80 years ago with some of the earliest publications on the subject issued in the early 1930s (30), while its crystallographic structure was determined in the early 1950s of the twentieth century (31). This coincided with the extensive development work on pressure transducers (32). Typically, two pressure calibration methods can be employed in the manufacturing facility (33): (1) using Aronson pressure generator (34) and (2) in the shock tube. However, to check the characteristic frequency of the sensor and its rise time in response to shock loading, only a shock tube can be used since loading rates of Aronson’s apparatus are insufficient for this purpose.

Typically, sensor calibration performed in the factory establishes maximum pressure and linear dependency between the applied pressure and voltage output from the sensor. Standard parts and equipment used for this purpose are hardly ever replicated in the laboratory, particularly if long signal cables are used. The differences between factory calibration setup and instrumentation used in the laboratory have to be checked to account for possible discrepancies in sensor response caused by impedance and triboelectric effects associated with cabling of different length. The methodology used in our lab relies on the measurement of the shock wave velocity which is related to pressure using Rankine–Hugoniot relationship. Figure 2B presents the discrepancies between factory calibration factors, and those obtained using the procedure developed in-house. For brand new sensors (number 9–18), the discrepancies are less than 7%; however, we must stress our method of calibration covers 0–60 psi range, which lies within practical interest for studying of blast TBI. Older sensors (3 years old, after hundreds of exposures), possibly due to wear and tear and some differences in manufacturing showed somewhat larger differences (sensitivity loss up to −10%) in calibration factors with two on the extreme end where the discrepancy was almost −40% (for sensors 1 and 2 in Figure 2B). We have not observed any consistent trends in the calibration factor differences, and it would appear it is a case-by-case occurrence. This observation, combined with a random occurrence of larger deviations in calibration factor values, necessitates this type of sensor evaluation, and justifies the additional time spent on these tests. For new sensors, the calibration is thus recommended before routine use, while experimentally observed loss of sensitivity and history of particular sensor should dictate the need for additional calibration.

### Sampling Rate

The sampling rate is an important experimental variable to capture the shape of the pressure profile. Since there are no universal measurement and reporting standards on shock wave parameters and data acquisition settings in the blast-induced neurotrauma (BINT) research field, a broad variety of sampling rates are typically reported, ranging from 10 kHz to 10 MHz (20, 24, 25, 28, 35–41). However, in a majority of publications, this parameter is omitted from reporting whatsoever, either because it was reported earlier in the original report on the characterization of the blast generating device, or simply because it is treated as a trivial test parameter. Not only is the sampling frequency important for capturing the shape of the pressure profile, it is also an important factor in optimizing the output file size, along with the number of recorded channels and acquisition time. Moreover, it is unknown what sampling frequencies are adequate to accurately capture the shock wave profile traveling at supersonic speeds. To address these questions and establish adequate thresholds, we have performed a series of experiments where three sampling frequencies were used: 10, 100, and 1,000 kHz (Figure 3). We used a single shock wave with a nominal intensity of 130 kPa in the test section (T4 sensor, Figure 1B). At first glance, there are no significant differences between the three sampling frequencies (Figures 3A–C), but differences have become obvious after quantification of three basic shock wave characteristics: rise time, peak overpressure, and impulse. The most significant changes are with respect to the rise time (Figure 3D): only at 1,000 kHz sampling rate it is captured accurately. Note, that time resolution at this frequency is 1 μs which is adequate to capture rise times on the order of 4–5 μs which are typical for moderate intensity shock waves (Mach number below 2) based on our experience to date (28). Interestingly, sampling frequency as low as 10 kHz still allows accurate capture of peak overpressure and impulse values. This is simply because in this case, the shock wave with 5-ms duration traveling at 400 m/s velocity is a 2-m long (head to tail) mass of compressed air traveling in the 6 m long shock tube. With this sampling frequency, 10 data points per millisecond will be captured resulting in 50 data points for the 5 ms duration, which appears adequate to capture pressure-time outline without significant errors. However, when shorter duration shock waves are of interest, i.e., below 1 ms, which is characteristic of shock waves generated outside the shock tube, the sampling frequency needs to be increased to more than 1 MHz (20, 41).

### Electrostatic Interference

**Figure 4:**
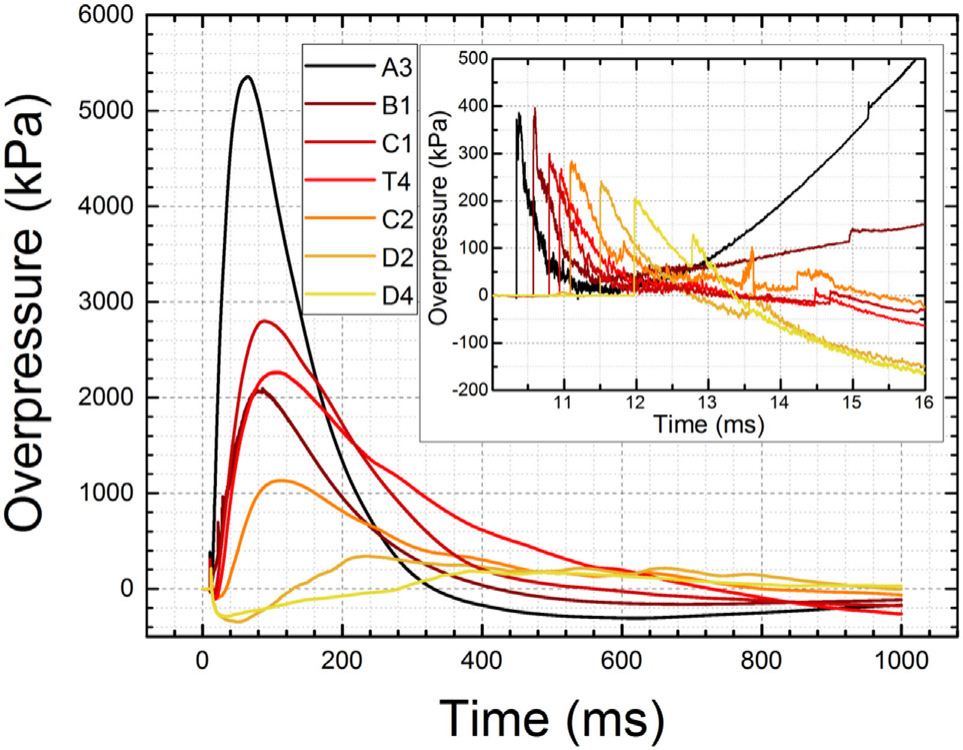
The baseline drift recorded by seven PCB 134A24 pressure sensors distributed along the shock tube (see Figure 1 for details). The data were recorded at 1 MHz frequency for 1 s (1 million data points per channel) for a shock wave with a nominal intensity of 250 kPa (a T4 sensor located in the test section). The burst pressure was 5,309 kPa (770 psi) for this test while erroneous peak pressure is 5,360 kPa for sensor A3 located at 1.43 m distance from the breech. The inset illustrates the shock wave profiles and baseline drift within 7 ms time frame. Note unrealistic negative pressure levels of −150 kPa reported by sensors D2 and D4.

We have performed more than 7,000 tests during the longevity of the shock wave testing laboratories at the University of Nebraska-Lincoln and then at New Jersey Institute of Technology. The shock tube walls have been painted to protect against rusting, but the downside is the paint also diminished dissipation of static electricity generated by the propagating shock wave. The PCB 134A24 tourmaline sensors mounted on the walls of both shock tubes for measurements of incident pressure suffer from a severe baseline drift which occurs as early as 1 ms after the shock front passage (Figure 4). The pressure reading reaches unrealistic values depending on the location of the sensor: nearly 5,400 kPa for the A3 sensor, and below −100 kPa for the D2 and D4 sensors; the unrealistic negative overpressure of less than 100 kPa are observed at 360 ms for sensors A3-T4. The maximum positive pressure for the A3 sensor is higher than burst pressure recorded for this test (5,200 kPa) and values of negative overpressure below −100 kPa are physically impossible to attain (vacuum pressure of outer space is below 10^−7^ kPa). These values demonstrate some other phenomenon is affecting pressure sensor readings. Considering extreme temperature variations on the order of hundreds of degrees are necessary to cause baseline drift in this class of pressure sensors, it leaves only electromagnetic radiation as the source of observed anomalous readings and baseline drifts. These baseline drifts while apparently not interfering with measurements of the shock front characteristics (rise time and peak overpressure) adversely affect the estimation of the shock wave duration and hence lead to the erroneous calculation of the impulse values. We have performed additional verification that the static electricity is associated with shock wave passage using a dummy sensor (Figure S1 in Supplementary Material). The voltage variations were observed and coincided with a shock wave initiation.

**Figure 5:**
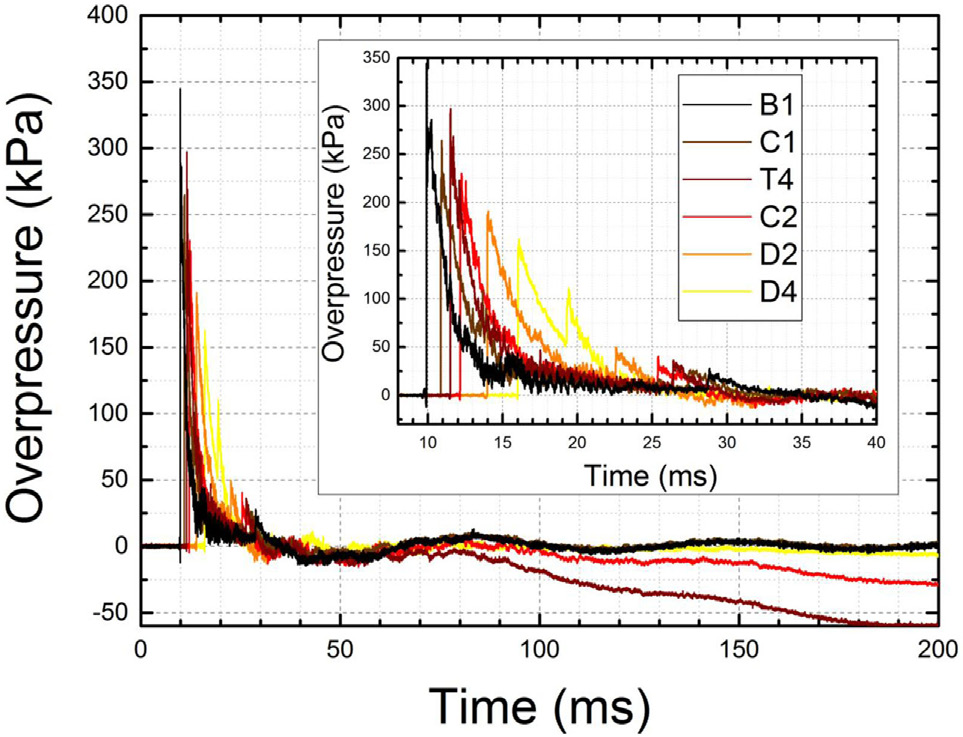
Baseline drift was eliminated with an aid of silicone insulator grease applied on the tip of the sensors. The baseline shows no signs of drifts toward positive or negative range over 200 ms of signal. Some baseline drift is observed for sensors T4 and C2, most likely due to gradual deterioration of silicone insulator layer caused by repeated exposure. The inset shows enlarged shock wave profiles recorded by six sensors for the nominal intensity of 250 kPa (T4 sensor).

The PCB Tourmaline Pressure Bars series 134A24 high-pressure, fast rise time sensors, with ranges up to 20,000 psi, are best suited for applications which require high-frequency measurements of the incident or reflected shock wave pressures like those found in studies of plasma physics and hypersonics. These sensors are factory protected against baseline drifts caused by high temperatures which exist near the epicenter of explosions or other high-pressure phenomena where heat is generated (42, 43), by a layer of a thermal insulator like vinyl tape. This method of sensor protection has proven ineffective against the level of static electricity generated in our shock tubes. We decided to resort to another compound with similar electrical insulation properties, commercially available silicone grease. Application of a thin 2–3 mm layer on the sensing surface of the sensors prevents baseline drifts and other adverse effects associated with static electricity as illustrated by overpressure profiles presented in Figure 5. It is obvious the baseline drift is virtually eliminated with this method. Periodic inspection and reapplication of the grease is necessary in order to record high quality, repeatable shock wave pressure profiles.

**Figure 6:**
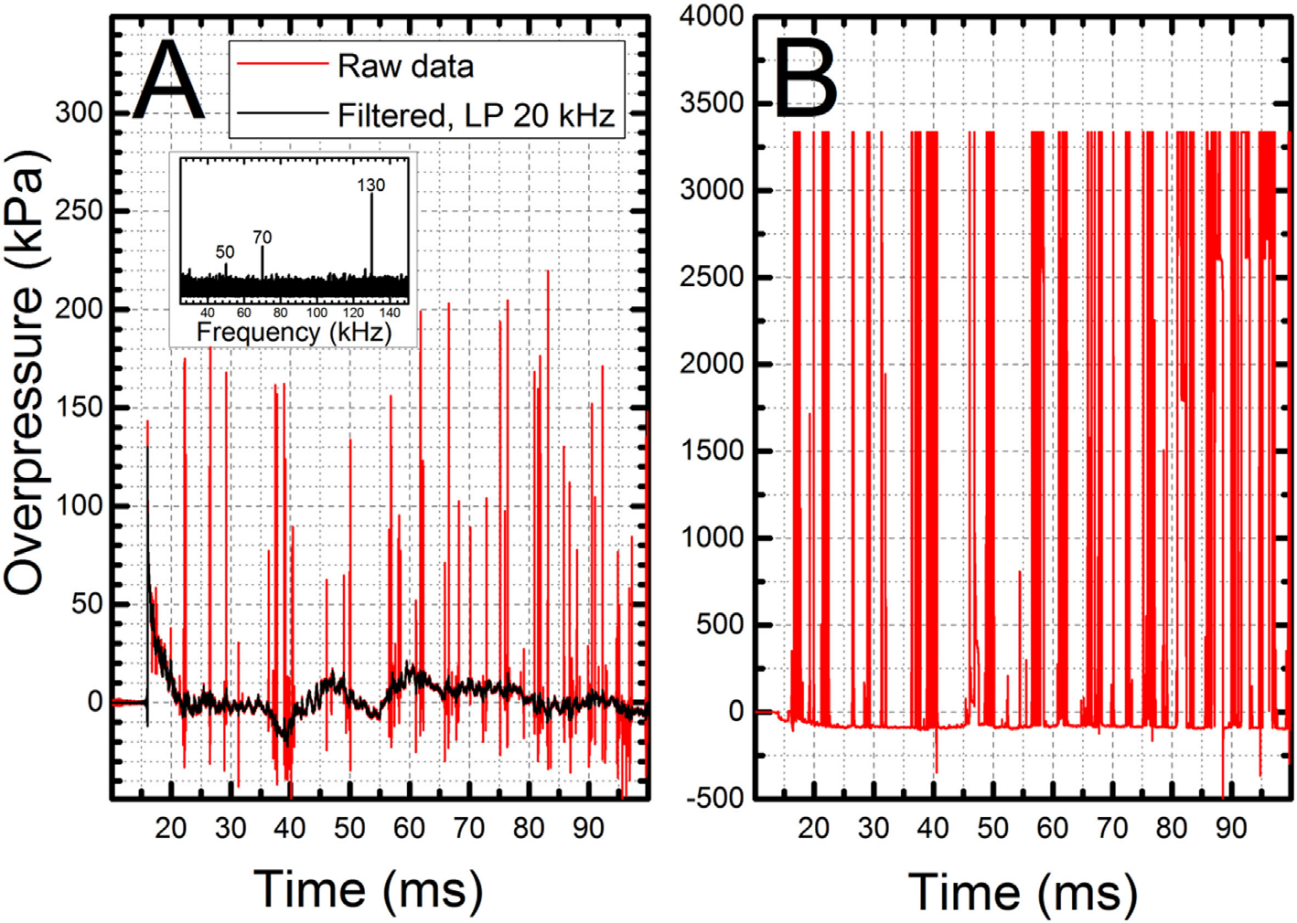
Electrostatic interference caused by breach of signal line insulation: **(A)** high-frequency components (inset) can be removed even from relatively contaminated data set using low pass (or equivalent) filter and **(B)** in extreme cases electrostatic interference reaches such levels the experimental data cannot be salvaged. This type of interference can be dangerous also for signal conditioners and DAQ modules due to sudden surges of DC currents *via* signal cables.

The second type of manifestation of the static electricity has become obvious during the testing of the effect of the shock wave loading conditions at different locations in the shock tube using the headform instrumented with PCB 102B06 (Figure 6B, inset). The sensor is connected to the low-noise data cable *via* 10-32 plug (PCB 002C10 low-noise cable) and then to the signal conditioner using a BNC connector *via* expansion terminal. Repeated exposure to the shock wave with 70–210 kPa intensity caused vibration of the headform which resulted in loosening of a connector between the pressure sensor and data cable. This in turn caused loss of electrical insulation of the data line running in the middle of the cable and results in a static electricity surge, i.e., a large number of high-intensity spikes randomly embedded in the pressure signal (Figure 6). Fast Fourier Transform analysis performed on this signal revealed three high-frequency bands: 50, 70, and 130 kHz. Due to relatively small amount of the signal contamination, in this case, it was possible to salvage the pressure data by running low-pass filter. However, in extreme cases, the loosening of 10–32 jacks resulted in unrecoverable levels of static electricity flooding the data channel (Figure 6B). The only remedy for this type of artifact is to secure the 10–32 plug with an electrical insulation tape to prevent the disconnection and a breach in electrical insulation.

### The Effect of Vibration on Pressure Signal Quality

**Figure 7:**
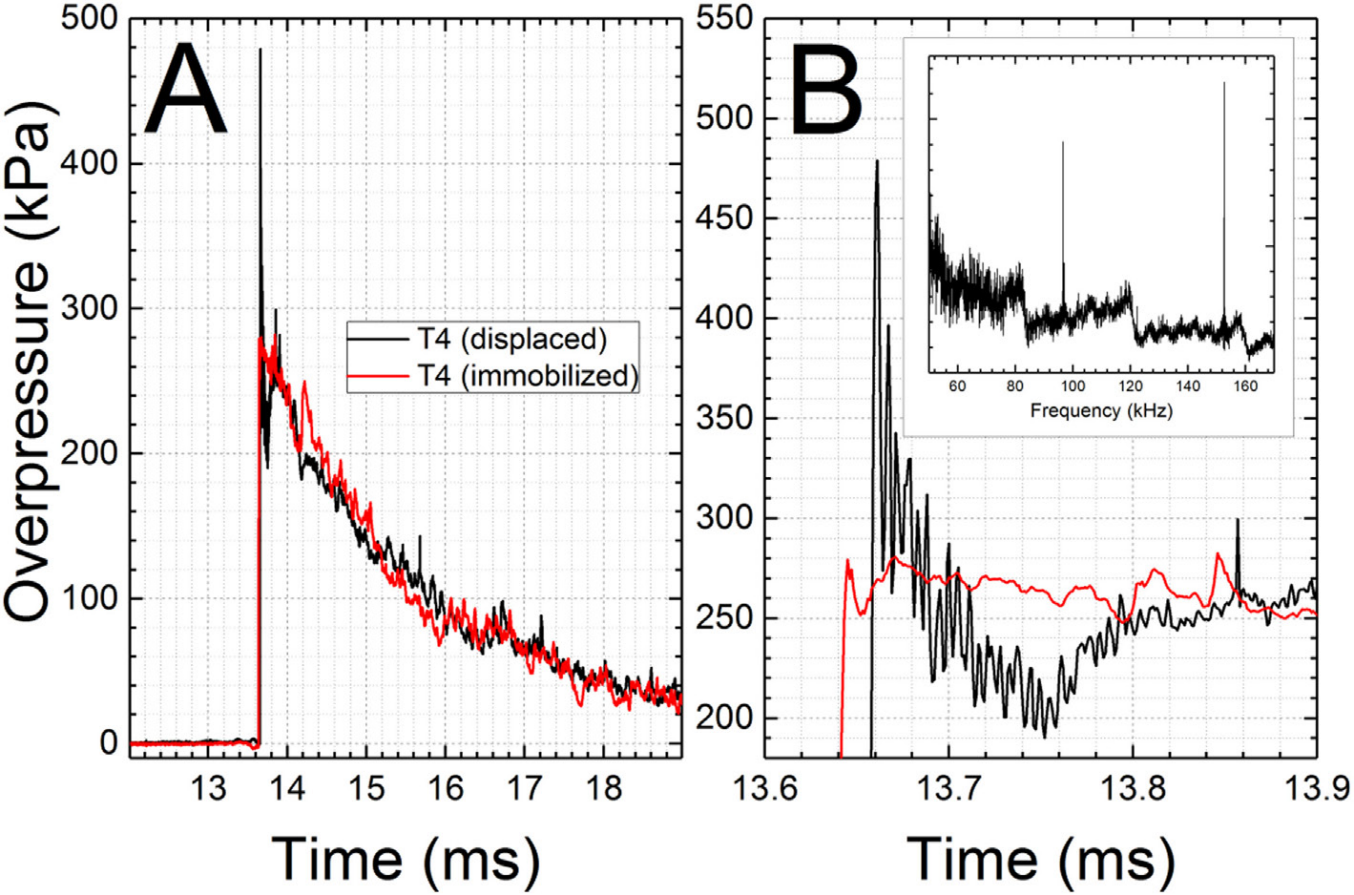
High-frequency signal observed in experiments where the sensor and the mounting bracket were pushed upwards by the passing shock wave. Comparison of the recorded overpressure with the artifact associated with the sensor [T4 (displaced)] and the signal with eliminated artifact **(A)**. Two brackets were used to prevent the displacement of the sensor resulting in clean waveform [T4 (immobilized)]. The magnified part of the pressure history reveals the presence of high-frequency noise and excessive peak pressure level **(B)**. Inset: FFT results of the pressure signal with artifact reveal characteristic frequencies: 96 and 152 kHz.

**Figure 8:**
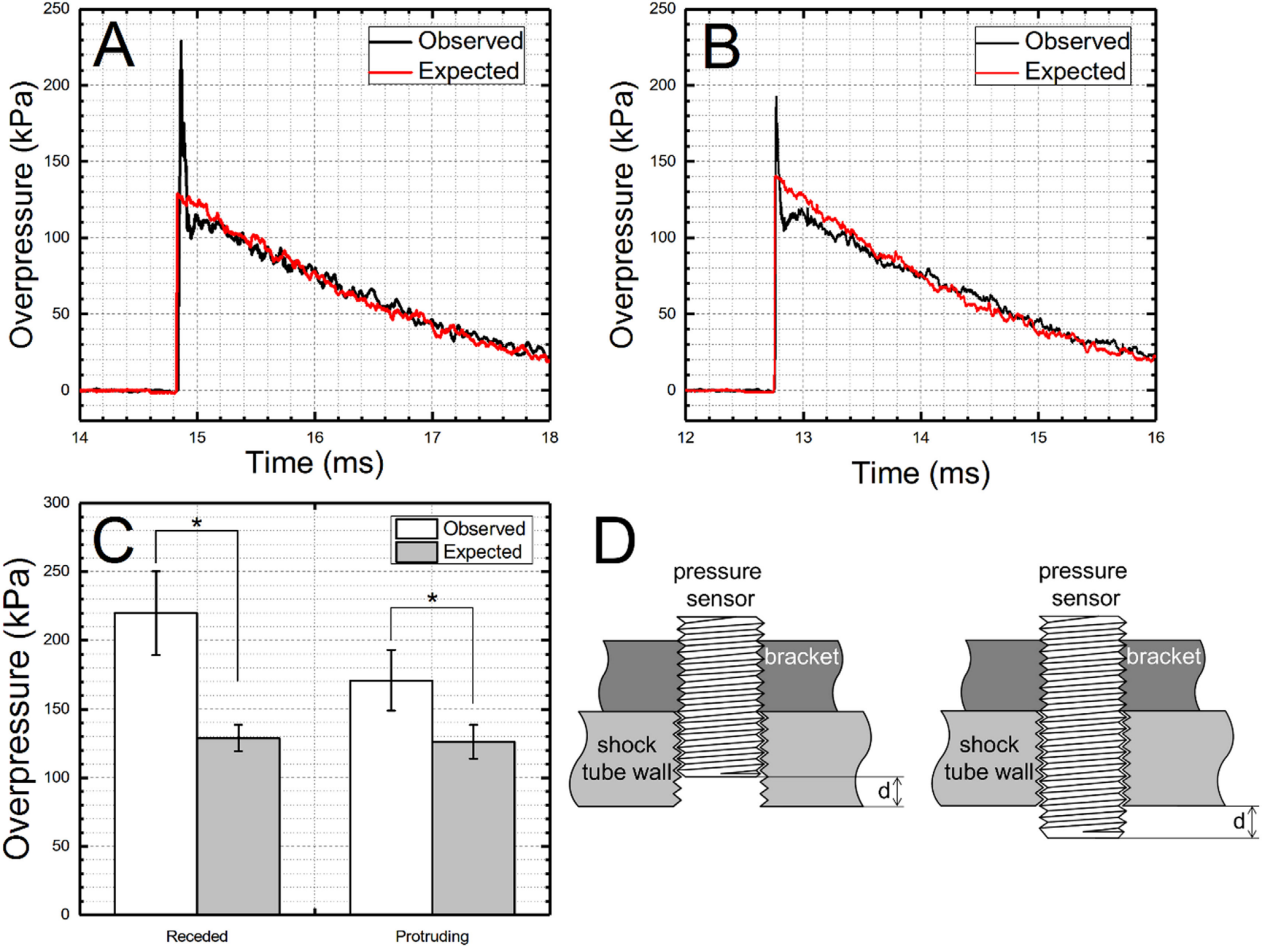
The effect of sensor misalignment with the shock tube wall on the resulting pressure profile. The exaggerated peak overpressure values are observed when the sensor is: receding **(A)** or protruding **(B)** from the shock tube wall. Quantification results (*n* = 4) for both tested scenarios demonstrate peak overpressure values are higher for receded sensor configuration than when the sensor was protruding **(C)**. These differences are statistically significant (*p* < 0.05). Nominal shock wave intensity of 130 kPa measured in the test section (T4 location) was used for these tests. Schematic representation of two sensor configurations resulting in peak overpressure artifacts is presented **(D)**. The distance between shock tube wall and sensing surface was the same in both cases (*d* = 0.25 in). Statistically significant differences are indicated with an asterisk (*p* < 0.05).

The peak overpressure is the only parameter consistently reported among shock wave characteristics (peak overpressure, rise time, duration, impulse, and sampling frequency). Sensor mounting and mechanical vibrations have the tremendous effect on the signal quality of the shock front and peak overpressure is an extremely sensitive parameter prone to misinterpretation. We observed sensors mounted on the top wall of the shock tube are being pushed upwards by the pressure of the passing shock wave: this is obvious on the high-speed videos (not shown) and the results in the corrupted initial part of the shock wave signal as seen in Figure 7. The upward motion of the sensor and its return to the resting position lasts only approximately 200 μs for the shock wave with 280 kPa peak overpressure (Figure 7B). The resulting signal is corrupted by the sensor translation within the opening of the mounting port. We have noticed this particular artifact present on numerous occasions in our data, and decided to install clamps on both sides of the pressure brackets to prevent the displacement of the bracket-pressure sensor assembly (Figure 1A, inset). However, to explore the origin of the exaggerated peak overpressure values in the controlled and systematic way, we decided to mount one of our sensors receded by 0.25 inch, and compare results with two other configurations where the sensor is mounted flush (default configuration for all experiments) and protruding by 0.25 inch (Figure 8). In both cases of non-flush sensor mounting, there is an excessive pressure buildup which lasts less than 100 μs (Figures 8A,B). Quantification of peak overpressure for *n* = 4 repeated trials indicates that the receded configuration results in a higher overestimation of peak overpressure (Figure 8C), and observed changes are statistically significant (*p* < 0.05). However, non-optimal sensor location cannot explain vibrations in the signal (Figure 7), which is likely associated with the impact of the sensor by the shock wave, and unrestricted displacement path while free-floating in the air. It would appear sensor vibration upon the initial shock wave impact leads to gross overestimation of the peak overpressure, while not affecting other characteristics. Literature survey related to reporting and existing standards of pressure measurements revealed some research groups were aware of the consequences of the improper sensor mounting. For example, Kochanek and co-workers mentioned in their recent work that “surface flush gauges were used for the shock measurements because it is known that a gauge protruding or recessed by less than 1 mm will affect measurements” (17). Examples of waveforms with a sharp spike at the beginning of the shock wave are scattered through the literature. Chavko and colleagues report that when they measured the pressure with a miniature fiber-optic sensor made by Fiso, they detected “a lower pressure spike than the spike measured with the PCB probe.” It would appear both sensors were not mounted perfectly flush, which was not recognized at the time and the difference in spike intensity was explained by mere differences in sampling rate, which was 40 kHz for Fiso sensor and 500 kHz for PCB sensors or by their specific characteristics (44). Similarly, the presence of high-intensity spikes in the reflected pressure measured at the end of the closed shock tube was interpreted as a peak reflected overpressure (45, 46), most likely incorrectly based on our experimental data (Figure 8).

However, while consequences of non-flush mounting are recognized, far less attention is dedicated to proper signal analysis and preventive measures when high-frequency artifacts are present in the signal. For example, a quick glimpse on the overpressure profiles published by Svetlov and co-workers reveals that in their experiments, this artifact leads to twofold overestimation of the peak overpressure [see Figure 2 in Ref. (47)], i.e., it was erroneously reported as 358 kPa, while, in fact, it was merely 180 kPa. Only in some instances where the sharp spike is present in the data correct peak pressure was reported, i.e., as the highest pressure resulting from the exponential decay part of the waveform (48).

## Conclusion

We have discussed two important experimental variables, mechanical vibration and electromagnetic interference on the quality of a waveform recorded using state-of-the-art high-frequency pressure sensors. The importance proper implementation of preventive strategies, understanding of pressure measurement artifacts, and data interpretation with examples provided in this paper will help the community at large avoid these mistakes. In order to facilitate interlaboratory data comparison on the BINT research area, common reporting standards should be developed. We noticed the majority of published literature on the subject limits reporting to peak overpressure, with much less attention directed toward other important parameters, i.e., duration and especially impulse, which should be included as mandatory in future contributions. We have demonstrated sampling frequency as low as 10 kHz can be used to capture correctly the shape of the shock wave with a few milliseconds duration. However, shock waves with shorter durations might require higher sampling rates, and a sampling rate of 1,000 kHz is typically necessary to capture the rise time, which might be an important parameter depending upon the design of the study. We have also demonstrated sensor calibration is a significant quality measure, particularly for sensors with an extended lifetime. While there were only slight differences between factory and in-house generated calibration factors, we must stress the importance of this step to obtain correct pressure values. It is extremely important in the recording of peak overpressure values, which seem to be very prone to errors.

## Author Contributions

MS and EA performed experiments and data analysis, MS and NC wrote the manuscript.

## Conflict of Interest Statement

The authors declare that the research was conducted in the absence of any commercial or financial relationships that could be construed as a potential conflict of interest.
