# Supplementary material for: On the Accurate Determination of Shock Wave Time-Pressure Profile in the Experimental Models of Blast-Induced Neurotrauma
Source: Front Neurol. 2018 Feb 6;9:52. doi: 10.3389/fneur.2018.00052 (PMC5808170; doi:10.3389/fneur.2018.00052)

**On the accurate determination of shock wave time-pressure profile in the experimental models of blast induced neurotrauma**

**Supporting information**

Maciej Skotak, Eren Alay, and Namas Chandra

^1^Department of Biomedical Engineering, New Jersey Institute of Technology, Newark, NJ 07102-1982, USA

**Table 1** Characteristics of the shock wave (peak overpressure, duration, impulse, rise time) and sampling frequency reported in 100 papers published between 1995 and 2017. Only papers on animal and in vitro models of blast TBI were selected with shock tubes or explosives as injury devices, using “blast TBI” keywords in Google Scholar search engine. The plus sign (+) indicates the property was reported, while empty cell indicates missing values. The total count is provided at the bottom row.

| **Year** | **Journal** | **DOI** | **Peak overpressure** | **Duration** | **Impulse** | **Sampling frequency** | **Rise time** |
| --- | --- | --- | --- | --- | --- | --- | --- |
| 1995 | Neuropath. And Appl. Neurobiol. | 10.1111/j.1365-2990.1995.tb01073.x |  |  |  |  |  |
| 1997 | Neuroscience Research | 10.1016/s0168-0102(97)01164-4 |  |  |  |  |  |
| 1999 | J Trauma | 10.1097/00005373-199907000-00023 | + | + |  | + | + |
| 2000 | J Neurotrauma | 10.1089/089771500415454 | + |  |  | + |  |
| 2000 | J Neurotrauma | 10.1089/089771500415454 | + | + |  |  |  |
| 2001 | J Trauma | 10.1097/00005373-200104000-00017 | + | + |  |  |  |
| 2001 | Brain Injury | 10.1080/02699050010009559 | + | + |  |  |  |
| 2004 | Crit Care Med | 10.1097/01.CCM.0000120051.79520.B6 | + |  |  | + |  |
| 2006 | J Trauma | 10.1097/01.ta.0000233742.75450.47 | + |  |  |  |  |
| 2006 | J Trauma | 10.1097/01.ta.0000233742.75450.47 | + | + |  | + |  |
| 2007 | Toxic. Sci. | 10.1093/toxsci/kfl138 | + |  |  |  |  |
| 2008 | J. Neurotrauma | 10.1089/neu.2008.0602 | + | + | + | + | + |
| 2009 | J. Neurotrauma | 10.1089/neu.2008.0748 | + |  |  |  |  |
| 2009 | J Neurotrauma | 10.1089/neu.2009-0898 | + | + |  |  |  |
| 2010 | J Trauma | 10.1097/TA.0b013e3181bbd885 | + | + |  |  |  |
| 2010 | J. Neurol. Sci. | 10.1016/j.jns.2010.04.010 | + |  |  |  |  |
| 2010 | J Neurosci. Res. | 10.1002/jnr.22510 | + |  |  |  |  |
| 2011 | J. Neurotrauma | 10.1089/neu.2010.1540 | + | + |  |  |  |
| 2011 | Neurobiology of Disease | 10.1016/j.nbd.2010.10.025 | + |  | + | + |  |
| 2011 | Frontiers in Neuroscience | 10.3389/fnins.2011.00042 | + |  |  |  |  |
| 2011 | Front Neurol | 10.3389/fneur.2011.00012 | + |  |  |  |  |
| 2011 | Front Neurol | 10.3389/fneur.2011.00019 | + | + |  |  |  |
| 2011 | J Neurotrauma | 10.1089/neu.2010.1513 | + | + | + | + |  |
| 2011 | Exp Neurol | 10.1016/j.expneurol.2011.09.018 | + |  |  | + |  |
| 2011 | J Neurotrauma | 10.1089/neu.2011.1990 | + |  |  |  |  |
| 2011 | J. Neurosc. Methods | 10.1016/j.jneumeth.2010.11.019 | + | + |  | + |  |
| 2011 | J Neurotrauma | 10.1089/neu.2009.1050 |  |  |  |  |  |
| 2011 | J Neurotrauma | 10.1089/neu.2010.1324 | + | + |  | + |  |
| 2011 | J Neurotrauma | 10.1089/neu.2010.1561 | + |  |  |  |  |
| 2011 | Neuroreport | 10.1097/WNR.0b013e328346b138 | + |  |  |  |  |
| 2011 | J Neurotrauma | 10.1089/neu.2009.1207 | + | + |  |  |  |
| 2011 | NeuroImage | 10.1016/j.neuroimage.2010.05.031 | + | + |  |  |  |
| 2011 | J Neuropathol Exp Neurol | 10.1097/NEN.0b013e3182189f06 | + |  |  |  |  |
| 2011 | J Neurotrauma | 10.1089/neu.2010.1532 | + | + |  | + |  |
| 2012 | Electrophoresis | 10.1002/elps.201200299 | + |  |  |  |  |
| 2012 | Electrophoresis | 10.1002/elps.201200319 | + |  |  |  |  |
| 2012 | Front Neurol | 10.3389/fneur.2012.00032 | + |  |  | + |  |
| 2012 | J Neurotrauma | 10.1089/neu.2011.2146 | + |  |  |  |  |
| 2012 | Magnetic Resonance Imaging | 10.1016/j.mri.2011.12.003 | + | + |  | + |  |
| 2012 | Front Neurol | 10.3389/fneur.2012.00111 | + |  |  | + |  |
| 2012 | J Neurotrauma | 10.1089/neu.2010.1591 | + | + | + | + |  |
| 2012 | J. Neurol. Sci. | 10.1016/j.jns.2012.02.002 | + |  |  | + |  |
| 2012 | Front Neurol | 10.3389/fneur.2012.00015 | + | + |  | + |  |
| 2012 | Hearing Research | 10.1016/j.heares.2012.01.013 | + | + |  |  |  |
| 2012 | Front Neurol | 10.3389/fneur.2012.00070 | + | + | + |  |  |
| 2012 | Rev Sci Inst | 10.1063/1.3702803 | + | + |  | + |  |
| 2012 | J Neurotrauma | 10.1089/neu.2012.2413 | + | + |  | + |  |
| 2012 | Front. Neurol. | 10.3389/fneur.2012.00046 | + | + |  | + |  |
| 2012 | Front. Neurol. | 10.3389/fneur.2012.00023 | + | + | + | + |  |
| 2012 | Sci Transl Med | 10.1126/scitranslmed.3003716 | + |  |  |  |  |
| 2012 | J Neurotrauma | 10.1089/neu.2012.2510 | + |  |  |  |  |
| 2012 | Front Neurol | 10.3389/fneur.2012.00177 | + |  |  |  |  |
| 2012 | Ann Biomed Eng | 10.1007/s10439-011-0420-4 | + | + |  | + |  |
| 2013 | Electrophoresis | 10.1002/elps.201300077 | + |  |  | + |  |
| 2013 | Neuroscience Letters | 10.1016/j.neulet.2013.07.047 | + |  |  |  |  |
| 2013 | Neuroscience | 10.1016/j.neuroscience.2013.08.037 | + | + |  | + |  |
| 2013 | PlosONE | 10.1371/journal.pone.0080138 | + | + |  |  |  |
| 2013 | Neuroscience | 10.1016/j.neuroscience.2013.09.021 | + | + |  | + |  |
| 2013 | J Alz Dis | 10.3233/jad-130182 | + | + | + | + |  |
| 2013 | J Neurotrauma | 10.1089/neu.2012.2773 | + | + | + |  |  |
| 2013 | Acta Neuropath Comm | 10.1186/2051-5960-1-52 | + |  |  |  |  |
| 2013 | Neurobiology of Disease | 10.1016/j.nbd.2012.12.002 | + |  |  |  |  |
| 2013 | J Neurotrauma | 10.1089/neu.2012.2758 | + |  |  |  |  |
| 2013 | J Neurosc Res | 10.1002/jnr.23179 | + | + |  | + |  |
| 2013 | Acta neuropathologica comm | 10.1186/2051-5960-1-51 | + |  |  |  |  |
| 2013 | Acta neuropathologica comm | 10.1186/2051-5960-1-51 | + |  |  |  |  |
| 2013 | Neurosci. Lett | 10.1016/j.neulet.2013.03.042 | + |  |  | + |  |
| 2013 | Exp Neurol | 10.1016/j.expneurol.2013.07.008 | + | + |  | + |  |
| 2013 | Neurobiology of Disease | 10.1016/j.nbd.2013.02.006 | + |  |  |  |  |
| 2013 | Neurosci. Lett | 10.1016/j.neulet.2013.01.028 | + |  |  |  |  |
| 2013 | Chem.-Biol. Interact. | 10.1016/j.cbi.2012.10.022 | + |  |  |  |  |
| 2013 | Ann Biomed Eng | 10.1007/s10439-013-0805-7 | + | + | + | + |  |
| 2013 | J. Neuroinflamm. | 10.1186/1742-2094-10-79 | + |  |  |  |  |
| 2013 | J Neurotrauma | 10.1089/neu.2012.2674 | + | + |  | + |  |
| 2013 | J Neurotrauma | 10.1089/neu.2012.2652 | + | + | + | + |  |
| 2013 | Free Rad Biol Med | 10.1016/j.freeradbiomed.2013.02.029 | + |  |  |  |  |
| 2013 | Front. Neurol. | 10.3389/fneur.2013.00154 | + | + |  | + |  |
| 2013 | Neurosci Lett | 10.1016/j.neulet.2013.03.042 | + | + |  | + |  |
| 2014 | Front. Cell. Neurosci. | 10.3389/fncel.2014.00421 | + |  |  |  |  |
| 2014 | Neuromol. Med. | 10.1007/s12017-014-8313-y | + |  |  |  |  |
| 2014 | Neurosci. Lett | 10.1016/j.neulet.2014.03.072 | + | + |  | + |  |
| 2014 | Mol. Cel. Neurosci. | 10.1016/j.mcn.2014.02.004 | + | + |  | + |  |
| 2014 | Shock | 10.1097/shk.0000000000000311 | + |  |  | + |  |
| 2014 | J Biomech Eng | 10.1115/1.4027873 | + | + | + | + |  |
| 2014 | Front. Neurol. | 10.3389/fneur.2014.00002 | + |  |  |  |  |
| 2014 | J Neurotrauma | 10.1089/neu.2013.3227 | + | + | + | + |  |
| 2015 | Mol. Neurobiol. | 10.1007/s12035-014-8902-7 | + |  |  |  |  |
| 2015 | J. Neurosci. Res. | 10.1002/jnr.23513 | + |  |  |  |  |
| 2015 | Front. Neurol. | 10.3389/fneur.2015.00132 | + | + |  |  |  |
| 2015 | Front. Neurol. | 10.3389/fneur.2015.00020 | + | + | + | + |  |
| 2015 | PlosONE | 10.1371/journal.pone.0127971 | + |  |  | + |  |
| 2016 | J Neurotrauma | 10.1089/neu.2015.3914 | + | + | + | + |  |
| 2016 | PlosONE | 10.1371/journal.pone.0161597 | + | + | + | + |  |
| 2016 | Sci. Rep. | 10.1038/srep26992 | + | + | + | + |  |
| 2016 | PlosONE | 10.1371/journal.pone.0167510 | + |  |  |  |  |
| 2016 | Exp Neurol | 10.1016/j.expneurol.2016.05.025 | + | + | + |  |  |
| 2016 | J Neurotrauma | 10.1089/neu.2015.3886 | + | + |  |  |  |
| 2017 | Sci. Rep. | 10.1038/srep41269 | + |  |  |  |  |
| 2017 | PlosONE | 10.1371/journal.pone.0173167 | + | + |  | + |  |
| 2017 | J Neurotrauma | 10.1089/neu.2015.4310 | + | + | + |  |  |
|  |  |  | **97** | **51** | **18** | **46** | **2** |

**Figure S1** Static electricity signal associated with a shock wave passage recorded using a dummy sensor. A piece of stainless steel tubing was isolated with a tape and inserted in the hole drilled in the bolt (A). The dummy sensor was mounted in the A3 port located 0.68 m before B1 sensor. Pressure profiles recorded by seven sensors and corresponding static electricity signal recorded by the dummy sensor (B).


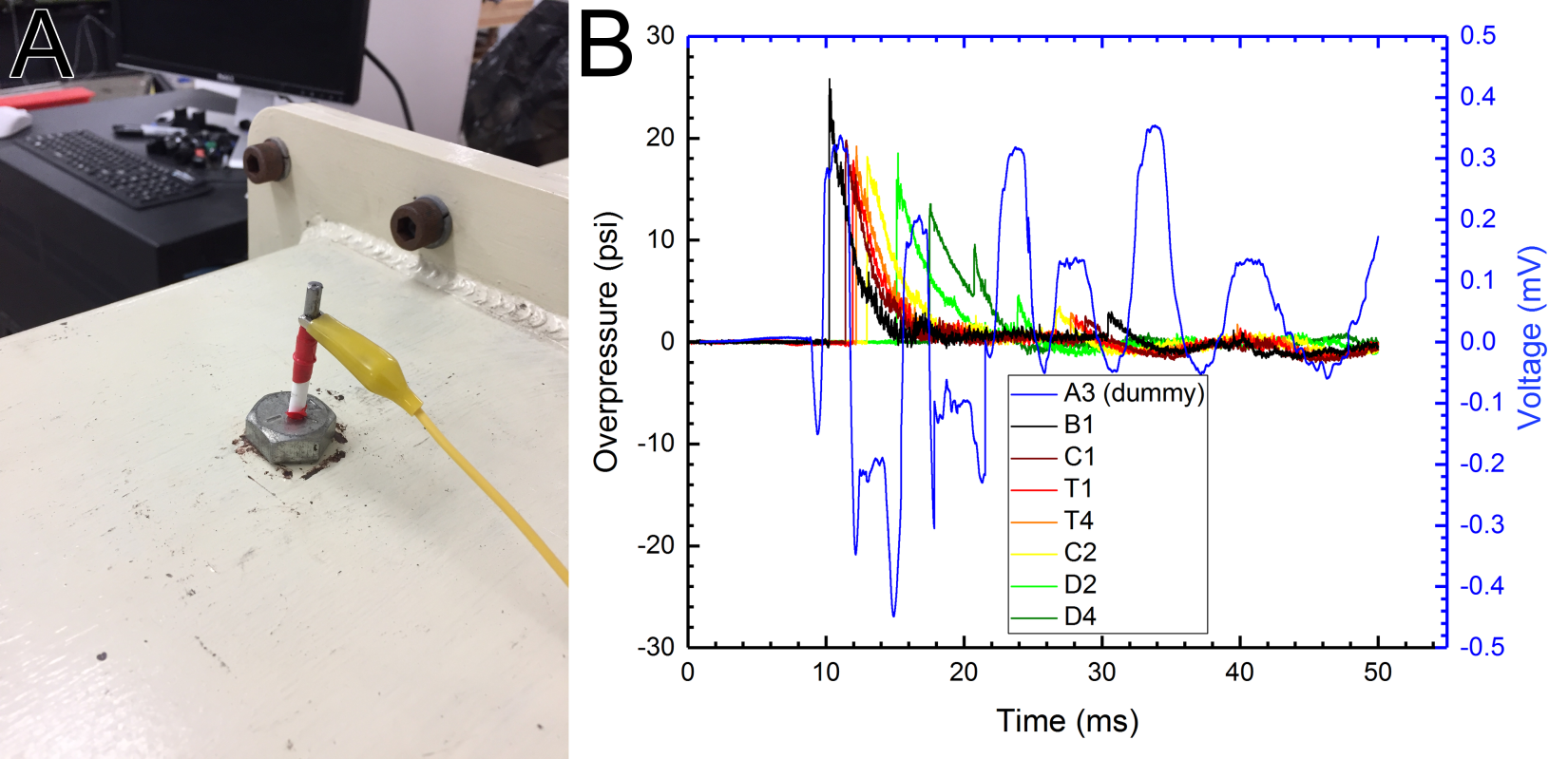

Supplement: Supplementary file 1 [file Table_1.docx]
